# Spatial Ecological Processes and Local Factors Predict the Distribution and Abundance of Spawning by Steelhead (*Oncorhynchus mykiss*) across a Complex Riverscape

**DOI:** 10.1371/journal.pone.0079232

**Published:** 2013-11-12

**Authors:** Jeffrey A. Falke, Jason B. Dunham, Christopher E. Jordan, Kristina M. McNyset, Gordon H. Reeves

**Affiliations:** 1 Department of Fisheries and Wildlife, Oregon State University, Corvallis, Oregon, United States of America; 2 Forest and Rangeland Ecosystems Science Center, U.S. Geological Survey, Corvallis, Oregon, United States of America; 3 Northwest Fisheries Science Center, National Oceanographic and Atmospheric Agency, Seattle, Washington, United States of America; 4 Pacific Northwest Research Station, United States Forest Service, Corvallis, Oregon, United States of America; Aberystwyth University, United Kingdom

## Abstract

Processes that influence habitat selection in landscapes involve the interaction of habitat composition and configuration and are particularly important for species with complex life cycles. We assessed the relative influence of landscape spatial processes and local habitat characteristics on patterns in the distribution and abundance of spawning steelhead (*Oncorhynchus mykiss*), a threatened salmonid fish, across ∼15,000 stream km in the John Day River basin, Oregon, USA. We used hurdle regression and a multi-model information theoretic approach to identify the relative importance of covariates representing key aspects of the steelhead life cycle (e.g., site access, spawning habitat quality, juvenile survival) at two spatial scales: within 2-km long survey reaches (local sites) and ecological neighborhoods (5 km) surrounding the local sites. Based on Akaike’s Information Criterion, models that included covariates describing ecological neighborhoods provided the best description of the distribution and abundance of steelhead spawning given the data. Among these covariates, our representation of offspring survival (growing-season-degree-days, °C) had the strongest effect size (7x) relative to other predictors. Predictive performances of model-averaged composite and neighborhood-only models were better than a site-only model based on both occurrence (percentage of sites correctly classified = 0.80±0.03 SD, 0.78±0.02 vs. 0.62±0.05, respectively) and counts (root mean square error = 3.37, 3.93 vs. 5.57, respectively). The importance of both temperature and stream flow for steelhead spawning suggest this species may be highly sensitive to impacts of land and water uses, and to projected climate impacts in the region and that landscape context, complementation, and connectivity will drive how this species responds to future environments.

## Introduction

Understanding habitat selection by an organism across landscapes represents one of the major challenges in contemporary behavioral ecology [1]. Landscape structure influences the behavior of organisms, which in turn generates patterns we observe in the distribution and abundance of individuals in local habitats [2]. Processes that influence habitat selection involve the interaction of landscape composition and configuration (e.g., [3]) across spatial extents encompassed by landscapes, particularly for species with specific habitat requirements and complex life cycles [4]. Common examples include amphibians which require complementary aquatic and terrestrial habitats to complete their life cycles [5], and birds which require distinctive and spatially discrete locations for feeding and breeding [6].

Although stream fish habitats are less commonly framed in the context of spatial ecological processes, many species have complex life histories and complementary habitat requirements [7], [8]. Spatial variability in habitat conditions across stream networks can produce spatially-structured populations [9], [10]. For example, recent work on Pacific salmon (*Oncorhynchus spp.*) in stream networks suggests that spatial patterns in the composition and configuration of habitat, not local habitat quality, drive patterns of spawning habitat use by Chinook salmon (*O. tshawytscha*; [11]). The challenge in considering spatial processes and their influence on fishes in stream networks lies in: 1) identifying the ability of individuals to move across the network relative to the distribution of available habitats, 2) constructing testable hypotheses about specific spatial processes at play based on the species’ dispersal ability, habitat needs, and spatial structuring of the stream network, and 3) quantifying local and spatial network processes in a way that allows for testing predictions derived from their hypothesized influences.

The combination of unique habitat requirements for different life stages, localized movement, and habitat selection suggests that salmonids should be strongly influenced by two key spatial processes: landscape complementation and neighborhood effects [3]. Landscape complementation refers to cases where an organism requires unique, non-substitutable, and spatially discrete habitats to complete its life cycle. Neighborhood effects refer to the influence of adjacent habitat conditions on an organism within a given focal location [12]. Because local and landscape features are inextricably linked, examining species distribution and abundance with reference to a spatially continuous representation of the composition and configuration of habitats within the stream network should contribute to a better understanding of the importance of spatial processes.

The well-known habitat requirements of salmon and trout [13] provide an ideal setting for testing predictions about the influence of spatial processes on species’ distributions and abundances. For salmonids, landscape complementation is a key spatial process involving use of a sequence of habitats through the life cycle (e.g., from egg to adult). Early life stages often disperse from natal habitats to neighboring locations that may be more suitable for growth and survival during rearing [14]. These localized movements are likely moderated by the dispersal ability of juvenile fishes, whose smaller body size and poor swimming ability, relative to larger adults, may limit their range of movement (i.e., extents ranging from 10^0^–10^1^ km; [15]). Intraspecific interactions (e.g., territoriality, dominance hierarchies) are strong during this period and may also influence movement [16]. Later in life, (one to several years, depending on species), juveniles can migrate long distances (10^1^–>10^3^ km) to reach complementary feeding habitats (e.g., large rivers, lakes, estuaries, or to the sea). Finally, adults migrate to reproduce, where natal homing and localized habitat selection ultimately determine spawning locations [17].

In this study, we developed continuous representations (i.e., covariates) describing processes hypothesized to influence the life cycle of steelhead (*Oncorhynchus mykiss*), an anadromous (marine-migratory) salmonid in a large river network. We tested whether predictors measured across spatial scales could explain the occurrence and abundance of steelhead redds (nests). The variables we considered were based on three simple requirements for individuals to successfully reproduce at a given location: 1) spawning grounds must be accessible; 2) reproduction needs to be successful; and 3) offspring must survive to continue the cycle [18]. We used geographic information systems (GIS) and count-based regression, combined with an information theoretic approach to: 1) develop spatially continuous representations of local, neighborhood, and landscape spatial ecological processes that may be important to the entire freshwater life cycle of steelhead; 2) evaluate the relative importance of these covariates in simultaneously predicting the distribution (i.e., occurrence) and abundance of redds; and 3) assess the predictive ability of models representing spatial versus local ecological processes. We evaluated our findings in light of the importance of interactions between complex life cycles, spatial ecological processes, and the distribution and abundance of organisms across heterogeneous landscapes.

## Materials and Methods

### Ethics Statement

Steelhead redd surveys were conducted by the Oregon Department of Fish and Wildlife (ODFW). No specific permits were required as surveys were strictly observational. No interactions with, or handling of, animals were conducted during sampling. Surveys were conducted on public and private lands. Landowner permissions were obtained prior to accessing privately owned stream reaches.

### Study System

**Figure 1:**
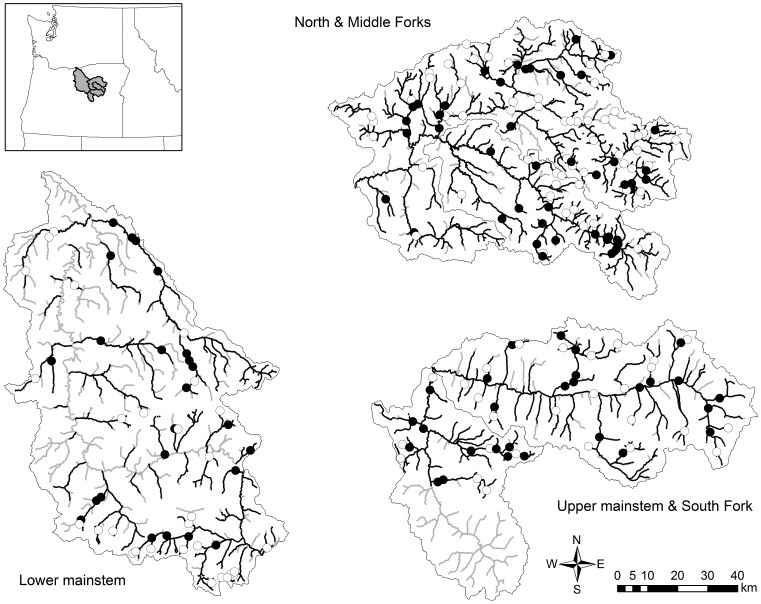
Map of the study area in the John Day River basin, Oregon, USA. Locations of 209 steelhead redd surveys conducted within five catchments (inset) from 2004–2010 are shown (circles). Filled circles are sites where redds were observed; redds were not observed at sites represented by an open circle. Bold stream lines indicate stream reaches that potentially support spawning and early rearing of steelhead (Oregon Department of Fish and Wildlife, *unpublished data*).

Our study area was the John Day River basin, located in eastern Oregon, USA (Figure 1). The John Day River is the second longest free flowing (i.e., undammed) river in the conterminous United States, draining approximately 20,000 km^2^, with a stream network comprised of over 15,000 stream km. Flow regimes vary across the basin with snow melt in most areas generating peak flows in spring, with base flows strongly augmented by groundwater in some locations [19].

Natural populations of steelhead in the John Day River are currently listed as threatened under the U.S. Endangered Species Act (ESA) owing to population declines caused by habitat alteration, hydropower, interactions with hatchery fish, and overharvest [20]. Steelhead in the John Day River basin consist of five naturally reproducing, genetically distinct spawning populations (hereafter referred to as TRT populations in reference to their designation by Technical Recovery Teams; [21]), which conform approximately to 5^th^ field hydrologic units (Figure 1; [22]).

### Field Sampling

Steelhead spawning surveys were conducted as part of ongoing status and trend monitoring by ODFW from 2004–2010. Sites were randomly selected using a generalized random tessellation survey (GRTS) design [23] from a sampling frame comprised of 4,269 stream km thought to support steelhead spawning (ODFW, *unpublished data*; Figure 1). The sampling frame was limited to exclude reaches above known natural and anthropogenic barriers to steelhead access (e.g., gradient barriers, waterfalls, culverts). Management goals were to sample approximately 50 2-km long sites per year. Sites were visited multiple times within the steelhead spawning season (February-June). During each visit, redds were visually identified from alongside the stream based on standardized protocols [24], their locations flagged, and new redd observations were recorded. Therefore, the total number of redds observed during a season at a survey site was the sum of the number of new redds across site visits. We assumed that sites containing more redds were more suitable spawning locations than those with few or no redds based upon the hypothesis that females should select sites with environmental conditions that maximize offspring survival. We took advantage of detailed temporal information within the redd survey data to calculate our response variable: the maximum number of individual steelhead redds observed at a site across years. We felt justified in using this approach for several reasons. First, the occurrence state of redds (i.e., present or absent) was temporally consistent. State changes occurred at only 4 of the 72 sites (6%) for which multi-year data were available. Second, counts were also temporally consistent among years at individual sites, as the average difference between maximum and minimum counts at a site was 2.34 redds (SD = 0.79). Last, we considered the effects of site length (km) and the number of years a site was sampled on redd occurrence and abundance. We found no relationship between site length and maximum redd counts via linear regression (see Results), or between presence and absence of redds and the number of years a site was sampled via logistic regression (see Results).

### Covariates

We used the FLoWS version 9.3 toolbox [25] for AcrGIS (ver. 9.3.1, ESRI, Redlands, CA) to create a digital hydrologic network for the John Day River basin based on the 1∶100000 scale National Hydrography Dataset (http://nhd.usgs.gov/). Values of covariates (described below) were attributed to stream reaches within the network at one of two spatial grains: 1) confluence-to-confluence stream reaches, hereafter referred to as valley segments (VS), or 2) 200-m reaches (200 M) nested within the valley segments. The 200 M reaches were pre-attributed with basic hydrologic and geomorphic information, including elevation (m), floodplain width (m), and drainage area (km^2^), which were used to calculate covariates [26].

**Figure 2:**
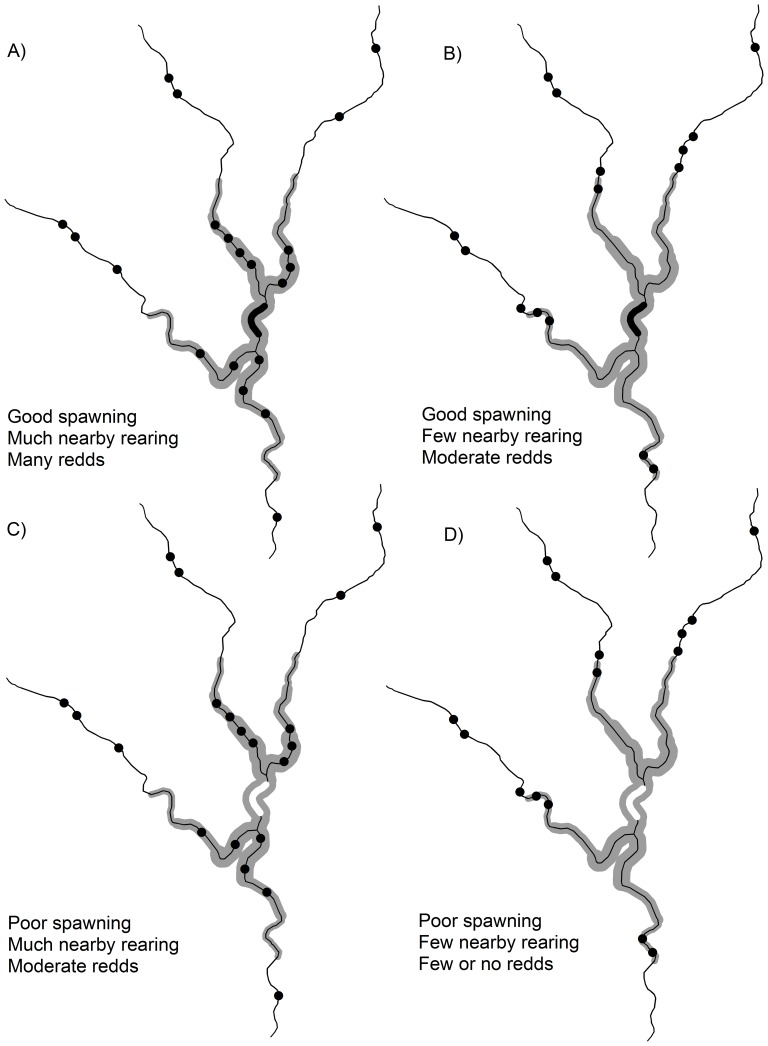
Conceptual model of hypothesized effects of landscape complementation on the occurrence of steelhead redds in stream networks. Likelihood of steelhead redd occurrence is indicated by black ( = high likelihood) or white ( = low likelihood) fill inside of the focal reach located within the center of each network. The likelihood of steelhead redd occurrence is hypothesized to increase when habitats utilized by juveniles (points) are abundant and located closer (along the stream network) to spawning reaches. Grey shading shows that the probability of juvenile movement exponentially declines with increasing stream distance from their natal reach (i.e., dispersal kernel).

Covariates were applied to stream reaches at two ecologically-meaningful spatial scales: within each 2-km long redd survey site, and for the “neighborhood” of stream reaches surrounding each site (hereafter referred to as local- and neighborhood-scales, respectively; Figure 2). Neighborhoods were delineated along the stream network using a recursive algorithm that measured the instream distance (km) from the midpoint of each site to every other 200 M reach within a pre-defined distance (A. Brookes, Western Ecology Division, U.S. Environmental Protection Agency, *unpublished data*). These neighborhoods encompassed all surrounding reaches including adjacent reaches and tributaries (Figure 2). We used a 5-km neighborhood size as a conservative estimate of juvenile steelhead dispersal distance [27]. We initially considered neighborhood sizes that encompassed 5 to 30 km, but found little difference in estimates of neighborhood characteristics summarized at the different distances.

We calculated seven covariates to represent key processes in the freshwater life cycle of steelhead that included: adult survival and spawning site accessibility, characteristics of the depositional environment, and juvenile survival. The first covariate we developed was a representation of stream size. Steelhead typically select spawning reaches across a broad range of mid-sized streams [28]. We characterized stream size as mean annual flow (MA; m^3^/sec) which was calculated using a variable infiltration capacity (VIC) macroscale hydrologic model developed for the Interior Columbia River basin [29].

Adult salmon and steelhead expend considerable energy while migrating from the marine environment to freshwater spawning locations and this influences adult pre-spawning survival [30]. We estimated the amount of energetic work (WORK) required by spawners to reach spawning sites as the distance from the site (midpoint) to the John Day River basin outlet (km) multiplied by the elevation (m) at the site. In the context of migration, distance represents travel time, and elevation is a surrogate for the average flow velocities encountered en route [31].

Locally, steelhead require suitably sized gravels for successful spawning [32]. However, at the neighborhood-scale the presence of suitable substrate may be correlated with habitat features (e.g., cover, pool depth, food resources) that influence the abundance and survival of offspring. As a result, females may choose spawning sites based not only on gravel size in the immediate area, but also on habitat potential for juvenile rearing within close proximity (Figure 2). In other words, if habitat in the surrounding neighborhood is of high quality a site may support more spawning than expected based only on local conditions. To address this hypothesis, we calculated median grain size (D50) based on methods developed by [33] (see Methods S1 for detailed methods of the D50 calculations). For our local-scale substrate metric (D50_SITE_), we calculated the proportion of reaches within a site that had an estimated D50 within the 25^th^ and 75^th^ percentile of substrate sizes used by steelhead (range 10–48 mm; [32]).

We also hypothesized that substrate characteristics within neighborhoods could influence selection of spawning locations (Figure 2). For our neighborhood metric, we calculated an index that weighted reaches based on their distance from the survey reach and their D50, using the sum of an exponential decay function: 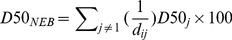
(1)where D50_j_ is 1 if D50 in reach *j* is between 10 and 48 mm, and 0 if not, and *d* is the distance between the midpoint of the survey reach *i* and the downstream end of reach *j*. In all cases D50 for reaches where redds were surveyed was excluded from neighborhood estimates.

Scouring flows are a significant source of mortality of eggs and fry through redd scour [34], and downstream displacement [35], respectively. As a result, females should avoid spawning at sites with a greater frequency of spring high flows that lead to a higher likelihood of scouring. We calculated a metric representing the frequency of high flows from Feb 1 to June 30 (S95) based on VIC model estimates [29]. Based on the results of the redd survey data, this period is arguably when redds are most susceptible to scour and fry are most likely to be displaced by high flows. The S95 was a count of the number of days that flows were in the top 5% within the Feb 1 to Jun 30 period.

Water temperature has strong influences on growth and survival of juvenile steelhead [36], and water temperature variability is an important component of aquatic habitat in the John Day River basin [37], [38]. We characterized the thermal regime for all valley segments in the John Day River basin by cumulative growing season degree days (sum of mean daily water temperatures (°C) from Jun 1 to Sep 31; GSDD). Mean daily water temperatures were estimated continuously across the John Day River basin using a riverscape temperature model (K. McNyset *unpublished manuscript*; see Methods S2 for detailed methods of the GSDD calculations). Preliminary analyses suggested that various temperature metrics at local- and neighborhood-scales (e.g., GSDD, mean summer temperature, etc.) were highly correlated (Pearson’s *r*>0.9), so to avoid issues with multicollinearity we included only GSDD in our models. The GSDD were estimated for each VS (i.e., 1–10 km scale), thus we considered it to be a neighborhood-scale predictor.

Finally, we included a categorical predictor (TRT) to identify the sub-population within which each site was located to explore among-population differences in patterns of redd abundance, and to account for residual spatial variation at larger (e.g.,>neighborhood) scales.

### Data Analysis

We used the “pscl” library in Program R to develop count-based regression models to predict the occurrence and the maximum number of redds at a site as a function of covariates measured at local and neighborhood scales. Specifically, we used a hurdle count regression model, which is a two-component model with a truncated count component for positive counts and a hurdle component that models the zero counts [39], [40]. The concept underlying the hurdle model is that a binomial probability model governs the binary outcome of whether a count variable has a zero or a positive value. If the value is positive, the “hurdle is crossed,” and the conditional distribution of the positive values is governed by a zero-truncated count model. This model structure allowed us to simultaneously model the probability of occurrence and the abundance of redds, and thereby investigate processes that lead to presence and absence of redds, and the number of redds observed at a spawning survey site. The binomial (occurrence) component was modeled using the binomial distribution with a logit link and the count (abundance) component with a negative binomial distribution with logit link.

We assessed the potential for multicollinearity among covariates using the variance inflation factor (VIF) statistic. Covariates with a VIF>10 were removed from analyses [41]. Covariates were log or arcsine-square root transformed as needed to meet normality assumptions of linear models. We also checked for problems associated with influential outliers by visually examining standardized residuals and for spatial dependency in model residuals (i.e., autocorrelation) using an empirical variogram (Figure S3; [42]).

We constructed a set of candidate models to represent biologically relevant combinations of processes measured at the local and neighborhood scales. Our candidate model set was formulated to represent alternative hypothesized effects of these processes on redd abundance, occurrence, or both (Table 1). We also considered a global model that included all terms. We then used an information-theoretic approach to find the most parsimonious set of independent variables to estimate the probability of occurrence and the maximum count of redds at a survey site [43]. We used Akaike’s information-criterion (AIC) to select the best approximating models by comparing candidate models. The AIC values were adjusted for small sample size (AIC_c_), and Akaike weights (*w*_i_) were calculated. The model with the lowest AIC_c_ and the highest *w*_i_ was considered the best model. To account for model uncertainty, we used model averaging to calculate parameter estimates and variances from models in the confidence model set (*w*_i_>0.05), and made inferences using this composite model.

**Table 1:** Candidate hurdle count regression models used to estimate occurrence and abundance of steelhead redds in the John Day River basin, Oregon.

Model^1^	Scale^2^	Hypothesis
MA^a^+WORK^a^+S95^b^+GSDD^a,b^+ D50_SITE_^a^+ D50_NEB_^a^+TRT^b^	Mixture	Global model
MA^a^+WORK^a^+S95^b^+GSDD^a,b^+ D50_SITE_^a^+ D50_NEB_^a^	Mixture	Global model without TRT
WORK^a^+GSDD^a,b^+ D50_NEB_^a^+TRT^b^	Neighborhood	Neighborhood+TRT
MA^a^+ S95^b^+D50_SITE_^a^+TRT^b^	Site	Site+TRT
WORK^a^+GSDD^a,b^+ D50_NEB_^a^	Neighborhood	Neighborhood-only
MA^a^+ S95^b^+D50_SITE_^a^	Site	Site-only
MA^a^+WORK^a^+TRT^b^	Mixture	Adult Survival/Access+TRT
S95^b^+ D50_SITE_^a^+ D50_NEB_^a^+TRT^b^	Mixture	Depositional Environment+TRT
GSDD^a,b^+TRT^b^	Mixture	Juvenile Survival+TRT
MA^a^+WORK^a^	Mixture	Adult Survival/Access only
S95^b^+ D50_SITE_^a^+ D50_NEB_^a^	Site	Depositional Environment only
GSDD^a,b^	Neighborhood	Juvenile Survival only

1 Covariates were applied to the occurrence (a) and/or abundance (b) model components.

2 Models were formulated to address hypotheses at two scales (Site and Neighborhood) and in combinations (Mixture).

### Model Diagnostics

We evaluated the predictive ability of the occurrence and abundance components of our hurdle model separately to evaluate their suitability for management applications (e.g., survey design optimization, prioritizing habitat restoration). For the occurrence component, we used the “PresenceAbsence” library in Program R to calculate the percentage of sites correctly classified (PCC; cutoff = 0.5) and area under the curve (AUC) statistics. Standard deviations were computed for each metric. As insufficient data were available for an independent evaluation dataset and subsequent cross-validation analysis, we used the “0.632+” bootstrap evaluation method [44], [45] to assess the predictive accuracy of our abundance model. This bootstrapping method uses an optimism estimate to adjust model evaluation statistics and provides a nearly unbiased estimate of the external predictive performance of the model. We used a range of evaluation statistics to assess different aspects of the abundance model predictive performance. Bootstrap estimates were calculated on observed vs. predicted redd counts using Pearson’s (*r*) and Spearman’s rank (*ρ*) correlations, average error, and the root mean square error (RMSE). The average optimism was calculated for each statistic across 200 replicate bootstraps.

## Results

Two-hundred and nine sites (Figure 1) were surveyed for steelhead redds across the seven year period (2004–2010). Of the 4,269 stream km within the sampling frame, 411 km (10%) were sampled at least once. Maximum redd counts at a site ranged from 0 to 17 redds (mean = 2.09, SE = 0.25; Figure S4), with redds being observed at 96 of the 209 sites (46%). On average, redd densities along these 2-km stream reaches were low. At the majority of sites where redds were observed, maximum counts were less than 10 redds (87%), and about half of occupied sites contained only 1 or 2 redds (47%). We found no relationship between sampling effort (site length and number of years a site was sampled) on either presence and absence of redds using multiple logistic regression (*F* = 1.54, *P* = 0.479) or maximum counts (>0) based on negative binomial count regression (*F* = 2.18, *P* = 0.827). As such, we did not account for these terms in our hurdle models. The model that included only neighborhood-scale covariates had the lowest AIC_c_ and the highest *w_i_*. Considerable model uncertainty existed as the site-only model and models that included the TRT covariate had *w_i_* values>0.05 (Table 2). However, models representing only a single aspect of the steelhead life-cycle, and the global model that included all terms, had essentially no support (*w_i_* <<0.05).

**Table 2:** Model selection metrics for hurdle count regression models fit to occurrence and abundance data for steelhead redds at 209 sites in the John Day River basin, Oregon.

Model^1^	K^2^	L-L	AIC_c_	ΔAIC_c_	*w_i_*
Neighborhood-only	7	−538.70	1091.5	0	0.413
Site-only	6	−539.90	1091.9	0.4	0.347
Neighborhood+TRT	11	−536.01	1094.2	2.7	0.110
Site+Hatchery	10	−537.87	1095.9	4.4	0.047
Global model without TRT	10	−537.87	1095.9	4.4	0.047
Global model	12	−540.76	1105.7	14.2	<0.001

1 Model results are ranked by AIC_c_ from best to worst, and Akaike weights (*w_i_*,)>0.05 are also shown.

2 *K* is the number of estimated parameters, L-L is the log-likelihood, and ΔAIC_c_ is the difference in AIC_c_ relative to the best model (see [41] for details).

### Occurrence of Redds

**Figure 3:**
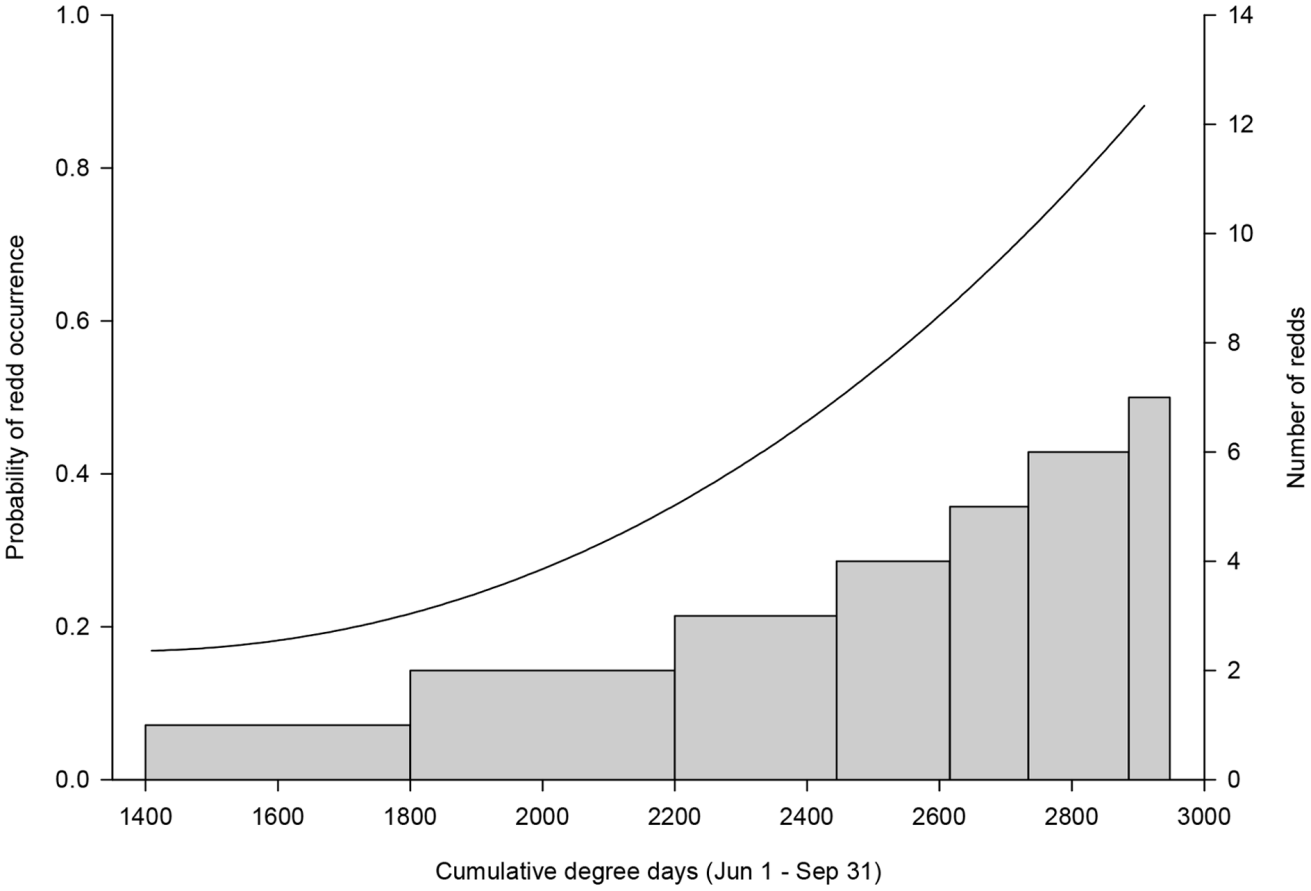
Probability of occurrence (line, left y-axis) and abundance of steelhead redds (bars, right y-axis) as a function of cumulative degree days (GSDD; °C) estimated from a hurdle count regression model. Horizontal thickness of bars indicates the approximate range of degree days predicted for a given count estimate.

**Figure 4:**
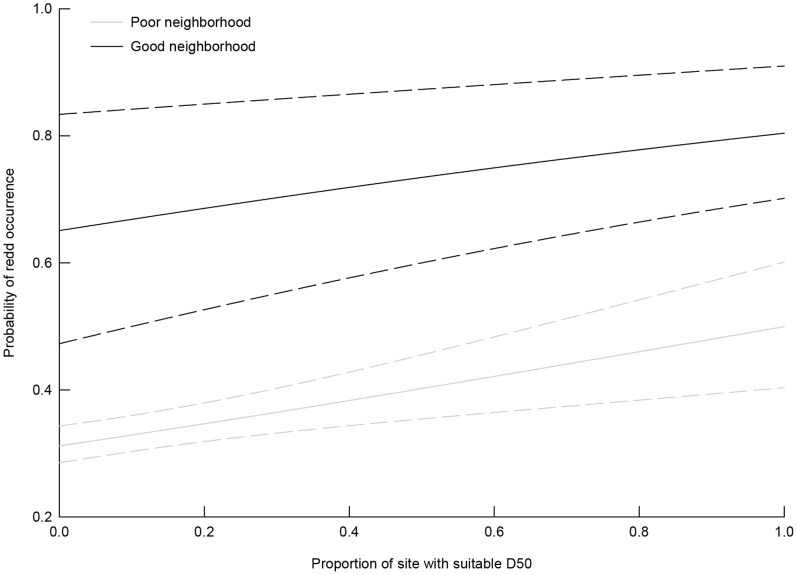
Probability of steelhead redd occurrence as a function of the proportion of a spawning survey site with suitable spawning substrates (D50_SITE_), in two neighborhood types : 1) Good has high amounts of suitable substrate in nearby reaches; and 2) Poor has low amounts. Estimates are from a hurdle count regression model. Dashed lines are 95% confidence intervals.

Results of the composite (i.e., model-averaged) model indicated that the probability of steelhead redd occurrence increased with: 1) stream size; 2) the proportion of suitable D50 within a site; 3) the amount of suitable D50 in close proximity to a site; and 4) growing season degree-days (Figure 3,Table 3). For a given proportion of suitable D50 within a site, the probability of redd occurrence more than doubled when availability of suitable D50 was greater in the surrounding neighborhood (Figure 4). Following model-averaging WORK was not significantly different than zero (i.e., confidence limits overlapped zero).

**Table 3:** Standardized model-averaged parameter estimates, unconditional SE values, and 95% confidence limits (CLs) for covariates predicting the occurrence (binomial model) and abundance (count model) of steelhead redds in the John Day River basin, Oregon.

	Covariate	Parameter estimate	SE	Lower 95% CL	Upper 95% CL
Occurrence model^1^	Intercept	−2.848	1.100	−4.993	−0.703
	MA	0.216	0.014	0.189	0.243
	D50_SITE_	0.243	0.019	0.207	0.280
	D50_NEB_	0.279	0.016	0.248	0.309
	GSDD	0.694	0.048	0.600	0.787
	WORK	−0.004	0.007	−0.018	0.010
Abundance model^1,2^	Intercept	0.240	0.021	0.198	0.282
	GSDD	0.683	0.063	0.560	0.806
	S95	−0.047	0.006	−0.058	−0.036
	TRTMF	0.014	0.086	−0.154	0.183
	TRTNF	−0.130	0.023	−0.174	−0.085
	TRTSF	0.000	0.061	−0.118	0.118
	TRTUM	0.029	0.094	−0.154	0.212
	Log(θ)^3^	−0.276	0.356	−0.970	0.419

1 Results are based on the top five hurdle count regression models, which were responsible for 96% of the collective model weight (see Table 2).

2 Levels for the TRT covariate are Lower Mainstem = Intercept, Middle Fork = TRTMF, North Fork = TRTNF, South Fork = TRTSF, and Upper Mainstem = TRTUM.

3 Log(θ) is the dispersion parameter.

### Abundance of Redds

The maximum number of redds at a site was predicted to be higher at sites with warmer thermal regimes (e.g., more GSDD), and lower at sites with more days of scouring flows (S95). At a given value of S95, expected redd counts varied across sub-basins even with GSDD held constant. For example, sites in the Lower Mainstem sub-basin close to the Columbia River were predicted to contain twice as many redds relative to sites in the North Fork sub-basin. Visual examination of the empirical semi-variogram (see Supplementary Materials) based on composite model residuals versus in-stream distance (km) indicated that the spatial pattern of the composite model residuals was not different from a random spatial pattern.

### Model Performance

Predictive performance of both the occurrence and abundance components of the composite model were generally better than the performance of either the non-averaged neighborhood- or site-only models (Table 4). The composite model PCC was 18% higher than the site-only model, but just 2% higher than the neighborhood-only model. The AUC statistic also was highest for the composite model, but differences among models were low. Redd abundance component evaluation statistics derived from bootstrap analysis indicated that the composite and neighborhood-only models performed best. Correlations between observed and predicted values for both Pearson’s and Spearman’s rank statistics were highest, and average model error was lowest, for the neighborhood-only model. Root mean square error was lowest for the neighborhood model (Table 4). Overall, the site-only model performed poorly in comparison to models that included neighborhood scale predictors.

**Table 4:** Model prediction diagnostics for three hurdle regression models predicting the occurrence (binomial) and abundance (count) of steelhead redds in the John Day River basin, Oregon.

	Occurrence^1^		Abundance^2^			
Model	PCC	AUC	*r*	*ρ*	AVE_error_	RMSE
Composite	0.80±0.03	0.90±0.02	0.79	0.74	1.17	3.37
Neighborhood-only	0.78±0.02	0.87±0.03	0.82	0.80	0.94	3.93
Site-only	0.62±0.05	0.81±0.04	0.66	0.62	2.44	5.57

1 For the occurrence component, percent correctly classified (PCC; cutoff = 0.5) and area under the curve (AUC) statistics with standard deviations are presented.

2 For the abundance component, the results of a “0.632+” bootstrap evaluation of Pearson’s *r*, Spearman’s *ρ*, average error (AVE_error_), and root mean square error (RMSE) of observed versus predicted redd counts are shown.

## Discussion

Our results emphasize the importance of spatial ecological processes that can drive habitat selection and ultimately patterns of presence and abundance at broad extents. More specifically, we found that selection of spawning locations by steelhead across a complex riverscape was strongly associated with habitats thought to provide complementary support to different life stages. Furthermore, habitat characteristics at both local and neighborhood extents were important in predicting the distribution and abundance of steelhead redds across a diverse riverscape. Landscape complementation is not often explicitly treated when modeling presence or abundance of a species [46]. We found two benefits to a more explicit treatment of complementation: a better understanding of spatial processes that influence a species, and improved prediction of species presence or abundance. In the case of steelhead, patterns we observed may be explained by both complementation and connectivity. Complementation was addressed specifically in our model by including metrics describing the quality of rearing and spawning locations near focal sites. Connectivity was implicitly factored into our predictive models by including a variable to account for the cost of travel to spawning locations as well as connectivity to reaches within a neighborhood. In other words, the synergistic effect of complementation and connectivity proved to be a powerful predictor of spawning locations and abundance of redds constructed by steelhead.

Landscape supplementation was not explicitly addressed in this study though it could also be important in predicting steelhead spawning locations and redd counts. Landscape supplementation is a mechanism by which organisms supplement resource acquisition by making use of identical resources located in nearby patches [3]. For example, a recent analysis of passerine birds showed that abundance was higher in natural locations where agriculturally-modified habitats provided supplementary feeding opportunities [6]. We suspect that landscape supplementation is likely to be important for steelhead as well, for analogous reasons. Landscape supplementation may provide redundancy in availability of key habitat requirements, thereby promoting persistence of steelhead in the face of environmental changes such as seasonal variability in flows and temperatures or episodes of major habitat rearrangements from catastrophic disturbances [47]. In practice it can be challenging to precisely describe and model a specific landscape process (e.g., complementation vs. neighborhood effects) that is clear and distinctive in concept ( [3], [48]; Figure 2). Regardless, a more explicit consideration of spatial ecological processes clearly improves our understanding of how species presence and abundance is expressed across broad extents.

The importance of spatial landscape processes to stream fishes has rarely been considered explicitly (but see [49]–[51]) despite being widely acknowledged [7], [10], [52]. Two explanations may account for this apparent discord. Spatial ecological processes are: 1) well-known, but not expressed within the rubrics of aquatic landscape ecology and 2) viewed inconsistently with respect to their relation to ecological scale (e.g., neighborhoods vs. metapopulations). However, there is little question that spatial landscape processes are implicitly acknowledged for stream fishes. For example, [53] found that the distance among spawning and rearing habitats was a fundamental factor explaining recruitment and population size of fishes in the Danube River. Moreover, the literature includes numerous references to distinctive life stages of stream fishes and their ecological requirements (e.g., [8]). However, more often than not these life stages and their requirements are studied in isolation, and thus connections among them (i.e., landscape complementation; see above discussion) are not clear (but see [10]). On the other hand, the importance of habitat connectivity is well-acknowledged and is the focus of intensive study (see review by [54]). Arguably, combining approaches to develop a more explicit treatment of connectivity should improve our understanding of stream fish ecology as evidenced by the results herein.

Predicting the distribution and abundance of organisms across complex landscapes is challenging as it requires accurately and precisely capturing habitat heterogeneity at scales that are relevant to the life history of the organism in question [55]–[57]. Past approaches to modeling effects of landscape configuration and composition on salmon and trout have relied on a patch-based approach [9], [11], [58], but relative to species in these studies, *O. mykiss* (i.e., steelhead and rainbow trout) is a habitat generalist that can occupy a wide range of habitat conditions [59]. The broad extent we addressed certainly encompassed multiple local populations of steelhead, but the boundaries delineating these populations were far from clear. Thus, we adopted an approach based on the concept of ecological neighborhoods that were delineated by the presumed movement behavior of steelhead. Unlike a patch-based approach, which is often focused on the importance of habitat size and connectivity, our neighborhoods were constant in size, but variable in their composition and connectivity. Whereas we were unable to evaluate the importance of neighborhood size or extent, we found that simply accounting for neighborhood effects dramatically improved our ability to predict presence and abundance of a generalist species. Given that many stream fishes are generalists [8] and that stream networks represent continuous gradients rather than discrete boundaries [60], [61], the concept of ecological neighborhoods seems widely applicable.

### Implications

Based on our rationale, what are some general guidelines for predicting the distribution and abundance of organisms with complex life cycles across large, heterogeneous landscapes? First, when spatial processes such as landscape complementation and neighborhood effects may be influential, an attempt should be made to measure covariates continuously, thus allowing the ecology of the organism to dictate the scale of the response. In an analysis such as the one we have proposed, failure to account for the habitat matrix may lead to biased estimates of population spatial structure [5], [62], [63]. Second, predictors should represent (spatial and behavioral) processes that are of primary importance in determining habitat suitability, not only at specific life stages but across the life cycle of an organism. These measures should incorporate knowledge of how individuals disperse across the landscape, select habitat in which to reproduce, and successfully produce offspring that survive to promote population persistence [64]. For our steelhead example we focused on access to spawning habitats, suitability of spawning gravels and the likelihood of disturbance of redds, and availability of conditions that subsequently support growth of offspring (i.e., thermal regime). We considered these factors to be of primary importance to persistence of steelhead in freshwater. Third, our results suggest that the effects of local habitat conditions should not be ignored. Although models that included local effects did not perform as well in a predictive sense as those that included neighborhood-scale measurements, we could not rule out their importance based upon model selection. We suggest that accounting for both neighborhood and local conditions are important, as based on the VIF analysis local habitat and neighborhood conditions were not good predictors of one another. Future work predicting steelhead spawning distributions could better characterize local-scale conditions by linking specific locations of redds (e.g., GPS coordinates) to individual reaches, thus providing information regarding the spatial arrangement (e.g., clumped vs. dispersed) of suitable reaches within a site. Estimates of substrate suitability at finer scales (e.g., channel units: pools, riffles, runs) might also improve predictive models that incorporate local-scale processes. Unfortunately, continuous mapping of geomorphic controls at very fine spatial grains remains difficult, although new technologies hold promise (e.g., LiDAR; [65]). Last, use of empirical data collected under a spatially balanced survey design, and statistical methodology that allows for the evaluation of alternative hypotheses are critical for an unbiased view of the influence of spatial ecological processes on the distribution and abundance of organisms in complex landscapes.

This study has numerous implications for evaluating and managing future climate impacts on steelhead. Changes in thermal and flow regimes are anticipated with regional climate warming [66]. We found that water temperature and stream flows most strongly influenced the distribution and abundance of steelhead redds. Climate change effects on the distribution of *O. mykiss* have only considered the resident form (i.e., non-anadromous rainbow trout) which appear to be the most resilient trout species with respect to climate change [19]. Whereas *O. mykiss* as a species may be resilient in the face of climate change, implications of climate change for life histories is another question. Expression of a resident (rainbow trout) or migratory (steelhead) pathway has been linked to thermal regime [67] and stream size [28], both of which were important in our models predicting the distribution and abundance of steelhead redds. More work is needed to better understand controls on migratory life history expression in this species, but climate-linked changes in life history expression seem quite plausible (see also [68]). Accordingly, the response of this species to changing climates will require an understanding of not only how climate affects stream flows and temperatures in both local and landscape contexts, but also how these factors may change the species itself.

In an applied context, the results of this work are generally relevant for species with complementary habitat needs, and have specific implications for conservation of steelhead, which is threatened across much of its range [20]. It is clear that presence and abundance of spawning locations were strongly tied to spatial landscape processes, namely connectivity, landscape complementation, and neighborhood effects. Quantifying these processes was possible using a combination of previously derived measures (e.g., VIC model output: MA, S95) and those generated by us (e.g., D50, WORK). The importance of these processes provides a fundamentally different view of why steelhead use specific locations for spawning. In essence, suitable characteristics of sites are a necessary but often insufficient condition for the persistence of steelhead populations. Accordingly, restoration of stream habitats can benefit from this broader perspective [69], [70]. For example, restoration of a specific site for a specific life stage may fail if steelhead are less likely to use the location due to limited connectivity, lack of complementary habitats for other life stages, or unsuitable neighborhood characteristics. In other words, the site itself is only one of several factors that will influence the outcome of a given restoration effort. Currently, there are many active efforts aimed at site-based restoration of salmon and trout habitat, but whether or not these efforts have resulted in positive biological responses is unclear. A major contributing factor is lack of suitable monitoring of these local efforts [71], but failure to consider spatial landscape processes is also a strong possibility [72]. Whereas active restoration at sites can produce short-term results, passive attempts to manage land and water uses at broader extents may be more successful in restoring natural landscape processes and fish populations over the longer term. Such an approach may be particularly important in the John Day River basin where recovery from widespread impacts of historical human activities will require decades [73].

## Supporting Information

Figure S1 **Empirical variogram** [**1**] **of residuals from a hurdle count regression model predicting the distribution and abundance of steelhead redds in the John Day River basin, Oregon, USA.** The variogram depicts the semivariance (y-axis) as a function of separation distance (x-axis) for 209 sites with 2.5^th^ and 97.5^th^ percentiles from 5000 permutations (dashed lines).
(DOCX)

Figure S2 **Frequency distribution of maximum steelhead redd counts in the John Day River basin, Oregon, USA collected from 2004–2010.**
(DOCX)

Methods S1 **Description of D50 and channel type classification methods.**
(DOCX)

Methods S2 **Description of methods for the water temperature model.**
(DOCX)
