# Supplementary material for: Spatial Ecological Processes and Local Factors Predict the Distribution and Abundance of Spawning by Steelhead (Oncorhynchus mykiss) across a Complex Riverscape
Source: PLoS One. 2013 Nov 12;8(11):e79232. doi: 10.1371/journal.pone.0079232 (PMC3827154; doi:10.1371/journal.pone.0079232)
Supplement: Figure S1 — Empirical variogram [1] of residuals from a hurdle count regression model predicting the distribution and abundance of steelhead redds in the John Day River basin, Oregon, USA. The variogram depicts the semivariance (y-axis) as a function of separation distance (x-axis) for 209 sites with 2.5th and 97.5th percentiles from 5000 permutations (dashed lines). (DOCX) [file pone.0079232.s001.docx]

FIGURE S1: SPATIAL DEPENDENCY IN MODEL RESIDUALS
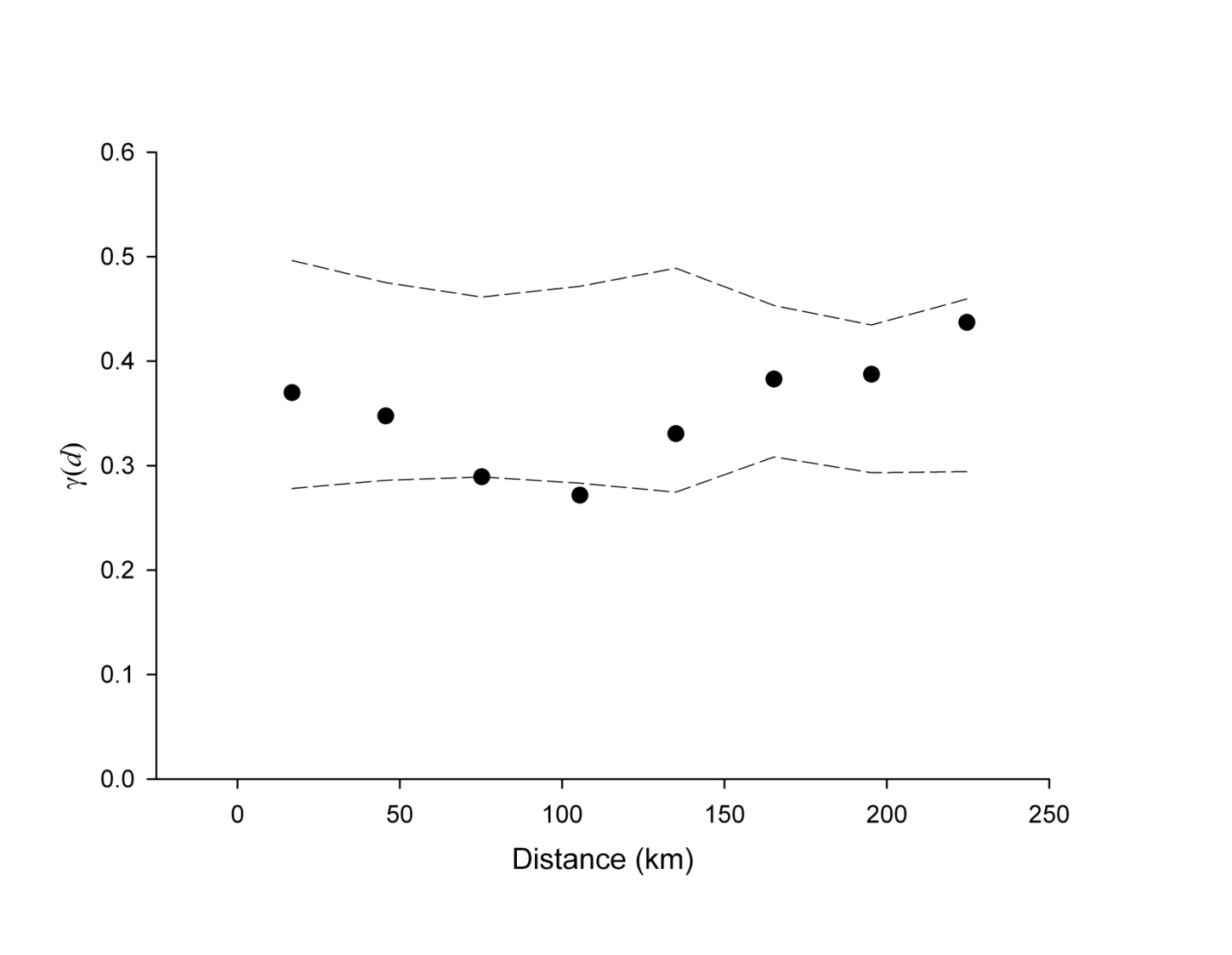


Empirical variogram [1] of residuals from a hurdle count regression model predicting the distribution and abundance of steelhead redds in the John Day River basin, Oregon, USA. The variogram depicts the semivariance (y-axis) as a function of separation distance (x-axis) for 209 sites with 2.5^th^ and 97.5^th^ percentiles from 5000 permutations (dashed lines).

1. Ganio LM, Torgersen CE, Gresswell RE (2005) A geostatistical approach for describing spatial pattern in stream networks. Front Ecol Environ 3: 138-144.
