# Supplementary material for: Spatial Ecological Processes and Local Factors Predict the Distribution and Abundance of Spawning by Steelhead (Oncorhynchus mykiss) across a Complex Riverscape
Source: PLoS One. 2013 Nov 12;8(11):e79232. doi: 10.1371/journal.pone.0079232 (PMC3827154; doi:10.1371/journal.pone.0079232)
Supplement: Figure S2 — Frequency distribution of maximum steelhead redd counts in the John Day River basin, Oregon, USA collected from 2004–2010. (DOCX) [file pone.0079232.s002.docx]

FIGURE S2: HISTOGRAM OF REDD COUNTS


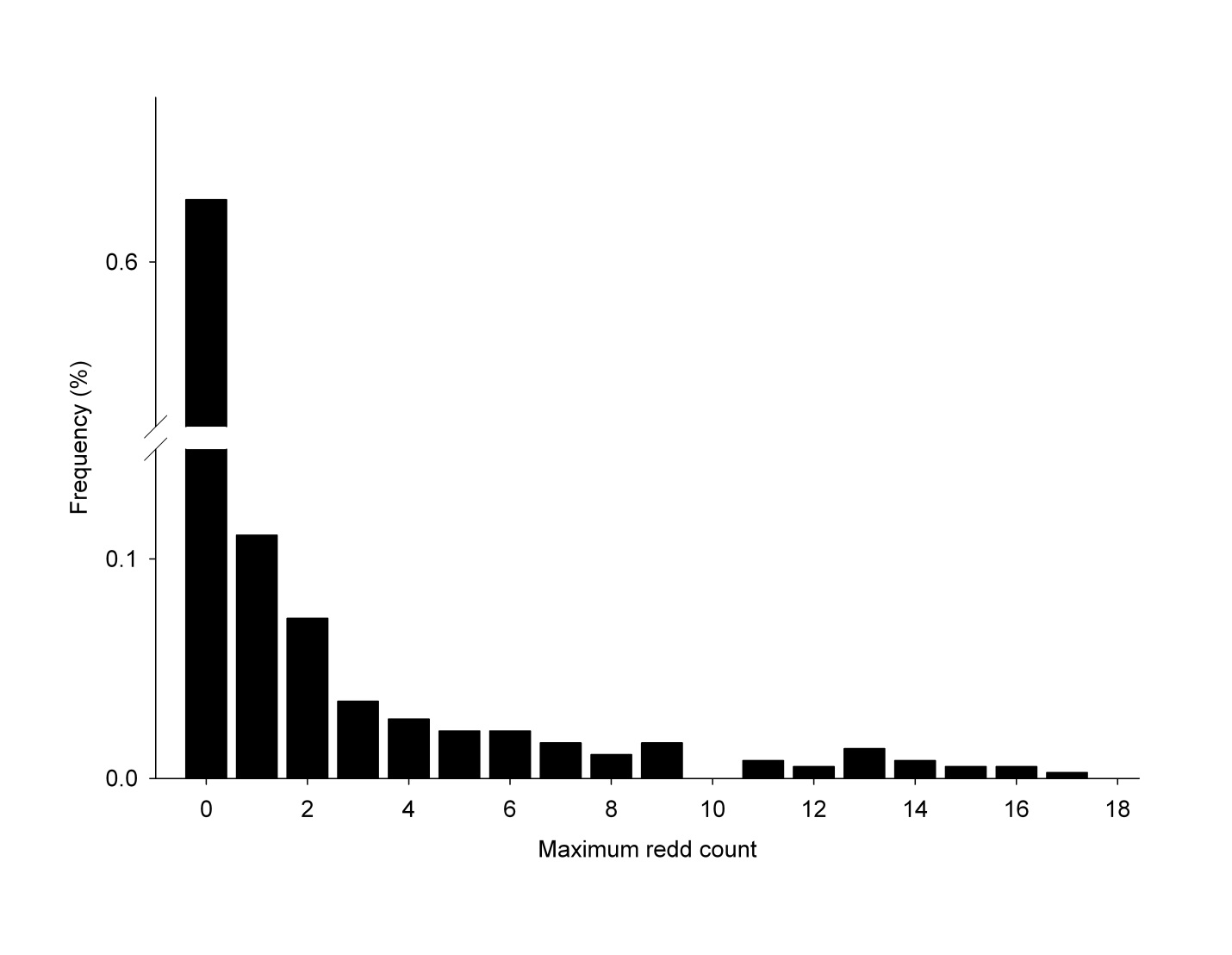


Frequency distribution of maximum steelhead redd counts in the John Day River basin, Oregon, USA collected from 2004-2010.
