# Supplementary material for: Spatial Ecological Processes and Local Factors Predict the Distribution and Abundance of Spawning by Steelhead (Oncorhynchus mykiss) across a Complex Riverscape
Source: PLoS One. 2013 Nov 12;8(11):e79232. doi: 10.1371/journal.pone.0079232 (PMC3827154; doi:10.1371/journal.pone.0079232)
Supplement: Methods S1 — Description of D50 and channel type classification methods. (DOCX) [file pone.0079232.s003.docx]

METHODS S1: D50 AND CHANNEL TYPE CLASSIFICATION

Owing to unique physiographic conditions among river basins, [1] suggested that local estimates should be generated for bank-full depth (*h*; m) vs. drainage area (*A*; m^2^) relationship and channel-type classification. Therefore, we first assembled empirical bank-full depth data from 94 sites located throughout the John Day River basin, collected during U.S. Environmental Protection Agency Environmental Monitoring and Assessment Program (EMAP) habitat surveys [2]. Next, we identified the 200M reach on which the mid-point of the EMAP survey was conducted and used the upstream drainage area to model *h* as a function of *A* using simple linear regression. We initially constructed regressions for each sub-basin (*basin*; see Fig. 2) in the John Day, under the premise that different relationships may exist across this broad physiographic region. However, we found no difference among the slopes or intercepts of the sub-basin scale relationships based on analysis of covariance (ANCOVA: *h* vs. *basin*, *F* = 1.04, *P* = 0.383, *h* vs. *A*basin*, *F* = 1.21, *P* = 0.314). As a result, we used the following relationship for the entire John Day basin (*F* = 64.21, *P* < 0.001, *r^2^* = 0.702):

$h= -1.37+0.129A$ (1)

Next, we classified each 200M reach to a channel type (Table 2). Our channel types were based on categories of channel slope (%) and drainage area (km^2^), validated from field estimates in the upper Middle Fork John Day (A. Przeszlowska, U.S. Forest Service, Olympia Forestry Sciences Laboratory, *unpublished data*). Finally, we estimated D50 for each 200M reach using equation 4 in [1], incorporating their bank-full Shields stress vs. bank-full shear stress relationships for each channel type. Colluvial channels occupy the most upstream, headwater portions of the channel network and typically have poorly sorted bed materials, are ephemeral or intermittent, and have low discharges. We did not calculate D50 for colluvial channels because we reasoned that steelhead do not use this type.

1. Buffington JM, Montgomery DR, Greenberg HM (2004) Basin-scale availability of salmonid spawning gravel as influenced by channel type and hydraulic roughness in mountain catchments. Can J Fish Aquat Sci 61: 2085-2096.
2. Kaufmann PR, Levine P, Robison EG, Seeliger C, Peck DV (1999) Quantifying physical habitat in wadeable streams. EPA/620/R-99/003. U.S. Environmental Protection Agency, Washington, D. C., USA.
