# Supplementary material for: Spatial Ecological Processes and Local Factors Predict the Distribution and Abundance of Spawning by Steelhead (Oncorhynchus mykiss) across a Complex Riverscape
Source: PLoS One. 2013 Nov 12;8(11):e79232. doi: 10.1371/journal.pone.0079232 (PMC3827154; doi:10.1371/journal.pone.0079232)
Supplement: Methods S2 — Description of methods for the water temperature model. (DOCX) [file pone.0079232.s004.docx]

METHODS S2: GROWING SEASON DEGREE-DAYS

This model was based on the relationship between observed mean daily water temperatures (°C) as a function of daily, remotely sensed land surface temperatures (K; LST). Mean daily water temperatures were compiled from water temperature loggers (*N* = 1757) deployed by state and federal agencies throughout the John Day River basin and covered the same time period as the LST data. Daily LST measures from NASA’s Moderate Resolution Imaging Spectroradiometer aboard the TERRA satellite (MOD11A1 Daily L3 Global 1km) were obtained through the online Data Pool at the NASA Land Processes Distributed Active Archive Center (<http://lpdaac.usgs.gov/get_data>). Subsequently, individual linear regression models were developed for each year during the period 2001-2009 using LST and Julian date as the predictor variables and water temperature as the response variable. Estimates were made for each valley segment within the John Day River basin. Modeled mean daily water temperature estimates for a given Julian date were averaged across years, and GSDD were calculated as the sum of the daily average temperature (°C) from 1 Jun to 31 Sep.
